# Evaluation of retinal vascular density and related factors in youth myopia without maculopathy using OCTA

**DOI:** 10.1038/s41598-021-94909-8

**Published:** 2021-07-28

**Authors:** Tiantian Wang, Hui Li, Rongrong Zhang, Yan Yu, Xin Xiao, Changfan Wu

**Affiliations:** 1 Department of Ophthalmology, Maanshan Shiqiye Hospital at Maanshan, Maanshan, 243000 Anhui China; 2 Department of Ophthalmology, Yijishan Hospital of Wannan Medical College at Wuhu, 92 West Zheshan Rd, Wuhu, 241000 Anhui People’s Republic of China; 3 Department of Ophthalmology, Wuhu Eye Hospital at Wuhu, Wuhu, 241000 Anhui China; 4 Visual Science and Optometry Center of Guangxi, People’s Hospital of Guangxi Zhuang Autonomous Region at Nanning, Nanning, 530000 Guangxi China

**Keywords:** Medical research, Eye manifestations

## Abstract

To evaluate the retinal vascular flow density changes of myopic eyes of young adults using optical coherence tomography angiography and the factors affecting these changes. In this cross-sectional study, 90 eyes of 45 participants were analyzed and divided into three groups: mild, moderate, and high myopia (without pathological changes). Macular and radial peripapillary capillary flow densities were measured using optical coherence tomography angiography. Their relationships with the axial length, the spherical equivalent of the refractive error, and age were analyzed using analysis of variance, Pearson’s correlation coefficient, and multivariate linear regression analysis. Superficial and deep macular vascular densities were significantly decreased in the high myopia group compared to the other groups. In the high myopia group, the nasal peripapillary flow density decreased, whereas the flow density inside the disc increased. The axial length negatively correlated with the superficial and deep macular vascular density, but positively correlated with the vascular density inside the disc. The spherical equivalent of the refractive error negatively correlated with the macular vascular density. The retinal vascular density decreased in the high myopia group. Hence, the microvascular network inside the disc may have a compensatory action in the hypoxic setting of high myopia.

## Introduction

Myopia is the most common ocular condition worldwide. The prevalence of myopia is 26.5% and 11.7% in adults and children, respectively. Myopia has a rapid growth trend, especially in Southeast Asia, as indicated by a survey conducted by the World Health Organization^1^. By 2050, it is estimated that 4758 million people (49.8% of the global population) will have myopia, a trend that will continuously increase the social burden^2^. Although it is currently believed that the number of outdoor activities and near work will play an important role in the pathogenesis of the disease, the cause of myopia remains unclear^3,4^. With an increase in the axial length, the possibility of retinal and choroidal complications of pathological myopia increases^5–7^. Several studies have indicated that vascular changes occur in high myopia and that these changes may be related to the pathogenesis of myopia^8,9^.

Optical coherence tomography angiography (OCTA), a rapidly developing technology introduced in 2014, permits the detection of blood flow through split-spectrum amplitude-decorrelation angiography algorithms, while reducing the noise due to the axial movement of the eyeball. AngioVue (Optovue, Fremont, CA, USA), an avascular imaging software, is used to display the flow of microvessels in the retina in a non-invasive way, thereby revealing erythrocyte movement. Due to its high axial resolution, OCTA enables the visualization of the retinal vasculature in multiple layers, and retinal and choroidal blood flow assessment without intravenous agents. Using this technique, a previous study showed that retinal perfusion is reduced in myopia, whereas the area of insufficient blood flow in the choriocapillaris is increased^10^. However, the participants have a large age range (25–83 years), a relatively small sample size (< 50 eyes); eyes with pathological myopia were included in the study concluding that retinal perfusion is decreased in high myopia. Elucidating whether the blood perfusion changes in the macular and optic disc areas of the myopic eyes of young adults changes before the pathological changes in the fundus and determining the factors influencing these changes will help understand the changes of blood flow in the macular area in the young Chinese myopic population, the pathophysiological mechanism of myopia, and find new ways for the prevention and intervention of myopia.

Therefore, this study aimed to observe the changes in the retinal vascular densities of myopic eyes of young adults using OCTA, and study the relevant factors that influence these changes.

## Results

We analyzed 90 eyes of 45 participants (12 males, 33 females). Table 1 shows no significant differences between mild myopia, moderate myopia, and high myopia groups in terms of sex and age; however, there was a statistically significant difference between the groups in terms of SE and axial length.

**Table 1:** Basic data of three groups of subjects.

Variables	Mild myopia	Moderate myopia	High myopia	*P* value*
No. of eyes	30	30	30	
Age (years)	28.57 ± 4.07	27.93 ± 6.36	28.33 ± 6.28	0.08
Boys, no. (%)	46.67	50	53.33	0.07
Spherical equivalent (D)	− 1.78 ± 1.1	− 4.33 ± 0.94	− 8.04 ± 1.46	** < 0.0001**
Axial length (mm)	24.20 ± 1.01	25.33 ± 0.82	26.66 ± 1.03	**0.01**

Significant *P* values are in bold.

In the high myopia group, the density of all vessels in the superficial macula and superficial parafovea was significantly lower than that of the other two groups (P < 0.0001 and P < 0.0001, respectively). In addition, there were statistically significant differences between the three groups regarding temporal, superior, nasal, and inferior vessel densities in the superficial parafovea (P < 0.0001) (with the lowest values in the high myopia group). In contrast, there was no statistical difference between the vessel densities in the superficial fovea (P = 0.40). However, there was a statistically significant difference between the mean deep total vascular densities and parafovea vascular densities (P = 0.01 and P = 0.02, respectively) of the groups; the mean vascular density of the high myopia group was significantly lower than that of the other two groups. There were also significant differences between the mean temporal, superior, and nasal deep parafovea vascular densities of the groups (P = 0.01, P = 0.02, and P = 0.04, respectively), with the lowest in the high myopia group. However, no significant differences were observed between the groups, regarding the mean deep foveal and inferior parafoveal vascular densities (P = 0.49 and P = 0.09, respectively) (Table 2).

**Table 2:** The vascular density in each area of the macula.

Variables	Mild myopia	Moderate myopia	High myopia	*P* value*
Superficial whole en face flow density (%)	51.76 ± 1.73	50.66 ± 1.96	48.83 ± 2.69	** < 0.0001**
Superficial fovea flow density (%)	19.08 ± 5.43	21.1 ± 7.32	20.88 ± 5.92	0.40
Superficial parafovea flow density (%)	54.36 ± 1.76	53.70 ± 2.06	50.85 ± 2.93	** < 0.0001**
Superficial temporal flow density (%)	54.35 ± 2.10	53.63 ± 2.01	50.88 ± 3.20	** < 0.0001**
Superficial superior flow density (%)	55.45 ± 2.16	54.72 ± 2.32	51.84 ± 3.45	** < 0.0001**
Superficial nasal flow density (%)	53.47 ± 2.35	53.05 ± 3.14	50.13 ± 3.34	** < 0.0001**
Superficial inferior flow density (%)	54.14 ± 2.32	53.42 ± 2.66	50.50 ± 4.07	** < 0.0001**
Deep whole en face flow density (%)	51.11 ± 3.56	50.96 ± 5.51	47.50 ± 6.03	**0.01**
Deep fovea flow density (%)	35.79 ± 5.65	38.10 ± 8.73	37.08 ± 7.67	0.49
Deep parafovea flow density (%)	56.77 ± 3.01	56.28 ± 4.26	53.90 ± 5.10	**0.02**
Deep temporal flow density (%)	58.18 ± 3.41	57.45 ± 4.06	55.10 ± 4.73	**0.01**
Deep superior flow density (%)	56.40 ± 3.51	56.17 ± 4.91	53.12 ± 5.67	**0.02**
Deep nasal flow density (%)	57.32 ± 2.67	57.28 ± 4.08	55.07 ± 4.70	**0.04**
Deep inferior flow density (%)	55.18 ± 3.39	54.32 ± 4.94	52.32 ± 6.41	0.09

ANOVA, analysis of variance.

Significant *P* values are in bold.

*All calculated by one-way ANOVA.

The vascular density in the radial peripapillary capillary (RPC) layer of the optic disc is shown in Table 3. The average RPC whole en face vessel density (N = 90) was 49.15 ± 2.66%. There were statistically significant differences between the three groups regarding the RPC whole enface vascular density, vascular density inside the disc, and peripapillary vascular density (P = 0.03, P = 0.006 and P = 0.03, respectively). The mean vascular density of the high myopia group was lower than that of the other two groups for the whole enface of the RPC and peripapillary layers but was higher inside the disc. In the high myopia group, the vascular densities of the upper and lower nasal areas around the optic disc were significantly reduced (P < 0.0001 and P = 0.006, respectively). There was no statistically significant difference between the three groups regarding the vascular densities of the inferior, temporal, and upper optic disc areas (*p* > 0.05).

**Table 3:** The vascular density of the RPC layer.

Variables	Mild myopia	Moderate myopia	High myopia	*P* value*
Whole enface (%)	49.83 ± 2.17	49.49 ± 2.49	48.12 ± 3.00	**0.03**
Inside disc (%)	53.83 ± 5.63	56.73 ± 3.92	57.30 ± 3.24	**0.006**
Peripapillary (%)	52.22 ± 3.13	51.13 ± 3.59	49.76 ± 3.94	**0.03**
Nasal upper (NU) (%)	48.97 ± 3.82	48.04 ± 4.41	43.95 ± 6.20	** < 0.0001**
Nasal lower (NL) (%)	47.45 ± 3.40	45.18 ± 4.72	43.58 ± 5.30	**0.006**
Inferior nasal (IN) (%)	50.72 ± 4.79	50.40 ± 4.62	48.09 ± 5.09	0.08
Inferior temporal (IT) (%)	57.31 ± 4.75	54.08 ± 9.11	55.10 ± 7.82	0.24
Temporal lower (TL) (%)	55.36 ± 3.34	53.03 ± 6.50	53.47 ± 6.03	0.24
Temporal upper (TU) (%)	56.12 ± 3.53	55.53 ± 4.96	56.43 ± 3.49	0.70
Superior temporal (ST) (%)	55.78 ± 3.70	55.64 ± 3.52	53.65 ± 5.60	0.11
Superior nasal (SN) (%)	50.8 ± 3.83	50.78 ± 4.14	48.84 ± 5.47	0.16

ANOVA: analysis of variance.

Significant *P* values are in bold.

*All calculated by one-way ANOVA.

**Figure 1:**
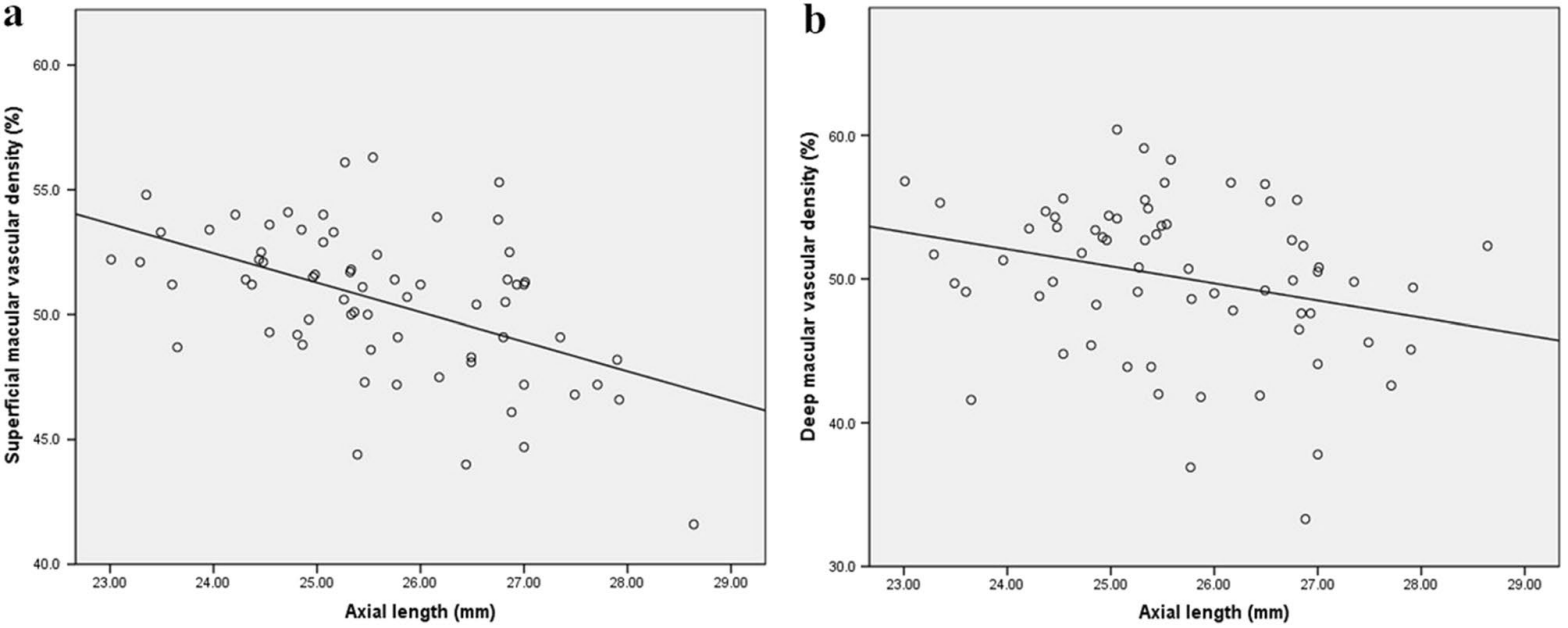
Scatter plots showing negative correlation between axial length and superficial and deep macular vascular density (**a**) superficial and (**b**) deep macular vascular density.

**Figure 2:**
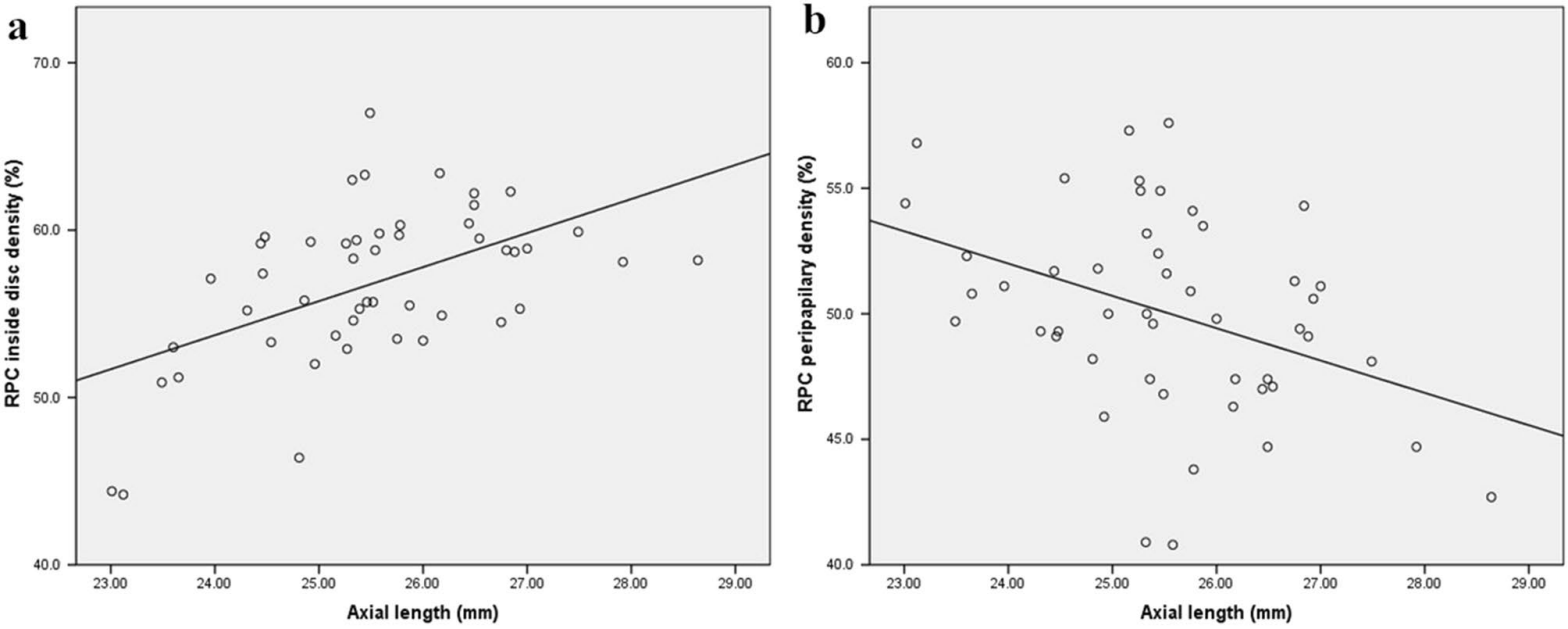
The association between axial length with vessel density inside the disc and in the papillary area (**a**) inside the disc, (**b**) the peripapillary area. RPC: radial peripapillary capillary.

**Figure 3:**
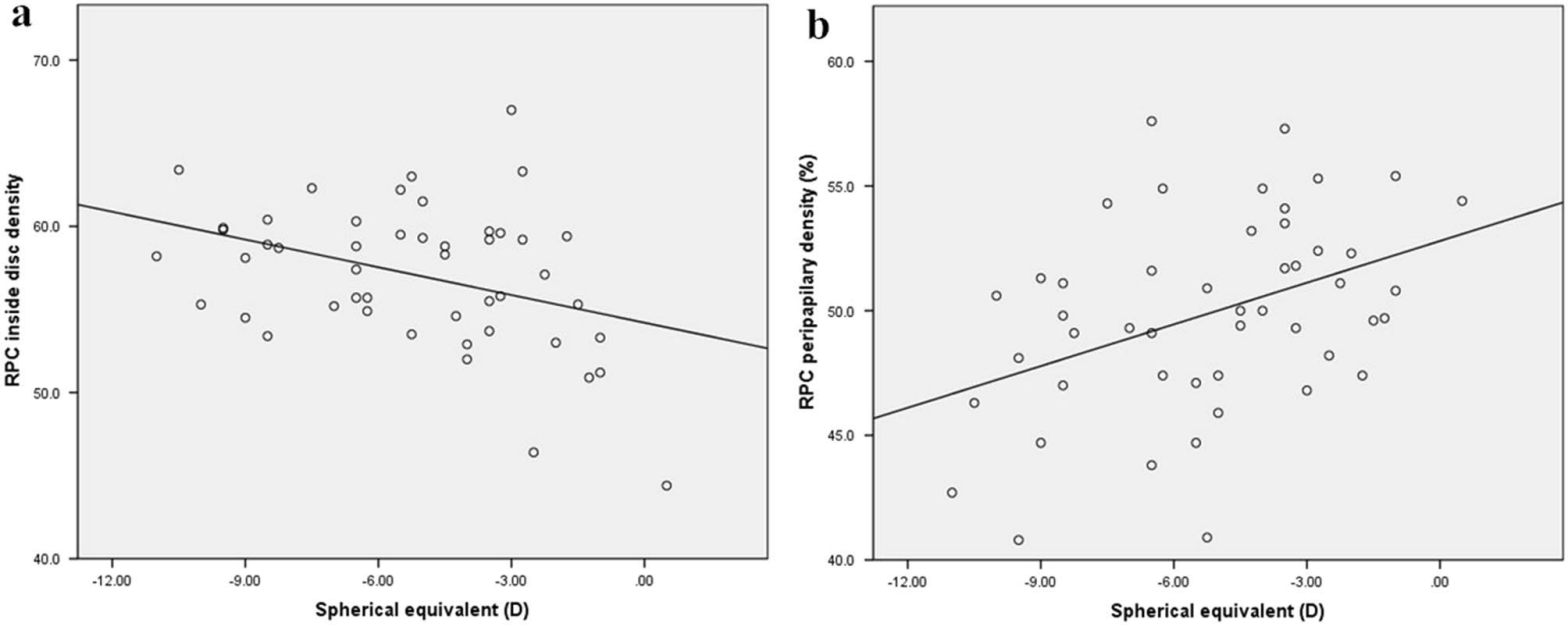
The association between spherical equivalent with vessel density inside the disc and in the papillary area (**a**) inside the disc, (**b**) the peripapillary area. D: diopter; RPC: radial peripapillary capillary.

Pearson’s correlation coefficient and linear regression analysis showed that superficial and deep macular vascular densities were significantly negatively correlated with axial length (r = − 0.500, P < 0.0001; r = − 0.272, P = 0.03; Fig. 1a,b, respectively); however, there was no significant correlation between age and spherical equivalent of the refractive error (SE) (P = 0.13, P = 0.53, P = 0.91, and P = 0.50, respectively). Furthermore, peripapillary vascular density was found to be negatively correlated with the axial length and positively correlated with the SE (r = − 0.386, P = 0.006; r = 0.408, P = 0.004; Figs. 2b, 3b, respectively), whereas vessel density inside the disc was found to be negatively correlated with the SE and positively correlated with the axial length (r = − 0.370, P = 0.01; r = 0.526, P < 0.0001; Figs. 2a, 3a, respectively), but had no significant correlation with age (P = 0.47, P = 0.18, respectively).

Multivariate linear regression analysis showed that the axial length had a strong negative relationship with macular vascular density in the superficial and deep layers (β = − 1.99, P < 0.0001; β = − 2.02, P = 0.02, respectively), and a positive relationship with the vessel density inside the disc (β = 1.74, P = 0.03) but not with the peripapillary vessel density (Table 4). In addition, there was a significant negative correlation between the SE and the superficial macular vascular density, even after adjustments for age, sex, and axial length (β = − 0.46, P = 0.01).

**Table 4:** Multiple regression analysis of associations with central foveal and the opic disc vascular density.

	Superficial foveal		Deep foveal		Inside the disc		Peripapillary	
	Estimate (95% CI)	*P*	Estimate(95% CI)	*P*	Estimate (95% CI)	*P*	Estimate (95% CI)	*P*
AL	− 1.99 (− 2.81 to − 1.17)	** < 0.0001**	− 2.02 (− 3.76 to − 0.27)	**0.02**	1.74 (0.16 to 3.33)	**0.03**	− 0.09 (− 1.56 to 1.38)	0.90
SE	− 0.46 (− 0.81 to − 0.11)	**0.01**	− 0.49 (− 1.24 to 0.27)	0.20	− 0.004 (− 0.65 to 0.64)	0.99	0.54 (− 0.06 to 1.14)	0.08
Age	− 0.03 (− 0.13 to 0.08)	0.63	− 0.14 (− 0.36 to 0.09)	0.24	− 0.09 (− 0.28 to 0.10)	0.32	0.13 (− 0.05 to 0.30)	0.15
Gender	0.48 (− 1.18 to 2.15)	0.56	1.74 (− 1.80 to 5.28)	0.33	1.37 (− 1.61 to 4.34)	0.36	0.28 (− 2.49 to 3.05)	0.84

AL: axial length; SE: spherical equivalent.

Significant *P* values are in bold.

## Discussion

In the present study, we analyzed the retinal vascular densities in myopic eyes of young adults. We found that the vascular densities in the superficial and deep macula and the nasal RPC layer were significantly lower in the high myopia group than in the other two groups. This finding is consistent with previous studies^11^. A key finding of the present study was that there was no statistically significant difference between the three groups regarding the vascular density in the fovea. This indicates that the SE did not affect on the vascular density in the fovea of the eyes without pathological myopic changes. In addition, we found that with an increase in axial length, the vascular density in the macular and peripapillary areas decreased significantly, and the blood flow inside the disc had a certain compensatory effect.

Before the widespread use of OCTA, various techniques were used to measure retinal blood flow. Doppler imaging was used to detect decreased retinal blood flow in the myopic eyes^9,12^. but it could not detect macular microvascular systems. OCTA was used to evaluate quantitatively macular vascular density, however, no significant difference between the foveal regions of the eyes with mild myopia, moderate myopia, and high myopia without pathological changes was observed. A possible reason is that the arterial blood supply is different in each part of the retina. Most of the fovea is an avascular zone characterized by active local metabolism^13^, maintaining the blood flow in a relatively stable state. Previous studies showed no significant difference in the foveal avascular zone (FAZ) area between myopic and control groups, consistent with our results^14,15^. Min et al., in contrast, concluded that the FAZ area was significantly larger in the myopic eyes^16^. Since different OCTA devices use different technologies and patients are of different ages, sexes, and ethnicities, it is not possible to standardize measurements. Our study used spectral-domain OCTA (RTVue XR Avanti, Optovue, Fremont, CA, USA), in which the border of the FAZ area was automatically positioned, manually adjusted. Another possible reason for this phenomenon is that our research participants were young people without myopic maculopathy. The age span was small; therefore, the change of FAZ was not obvious.

As the avascular zone extends outward, blood vessels begin to appear in the parafoveal area and the vascular density changes accordingly. In myopic eyes, excessive elongation of the eyeball may result in thinning of the retina, reducing the need for oxygen and resulting in reduced blood circulation. This subsequently causes the decreased blood flow density in the macular area. However, Wang et al. reported that the parafoveal vascular perfusion in myopic and emmetropic eyes was not significantly different^17^, possibly because they included younger participants in their study and because the early changes that occur in myopic eyes did not occur in the parafoveal regions of the included eyes; thus, the effect on the vascular density in the parafovea was not significant. The average age of the participants in their previous study was between 16 and 17 years, whereas the participants in the present study were between the ages of 20 and 40 years. This disparity in the ages of the participants led to inconsistent results. In addition, the changes in the macular vascular densities of myopic eyes and the pathophysiological mechanism behind them are not clear. Future studies with larger sample sizes and basic and clinical research with multi-directional objectives are needed to clarify and understand the facets of this mechanism.

In this study, we observed a significant reduction in the vascular density of the peripapillary area of highly myopic eyes, especially in the upper nasal region, and increased flow density inside the disc. Previous studies have demonstrated that decreased perfusion of the blood flow index and vascular density occurs in the peripapillary capillary network layer of highly myopic eyes. This finding that is consistent with our results^18^. We also observed a significant decrease in retinal flow density on the nasal side. Some researchers believe that the retinal nerve fiber layer thickness positively correlates with the peripapillary perfusion parameters^17^. The thickness of the temporal retinal nerve fiber layer increases with an increase in the axial length^19^. It is speculated that this effect might be due to the redistribution of the retinal nerve fiber layer. With an increase in the axial length, the retina is dragged toward the temporal horizon, the thickness of the temporal retinal nerve fiber layer increases, and the nasal side becomes thinner^20^. However, we did not observe an increase in the vascular density around the temporal peripapillary area; thus, further research is needed to verify this finding. Another important finding in the present study was that the vascular density inside the optic disc was higher in the highly myopic group than in the other groups. This finding can be explained by the fact that retinal vascular regulation becomes more significant through an automatic adjustment mechanism. As decreased peripapillary vascular perfusion may affect regional oxygen demand, increased blood flow inside the disc can alleviate the decreased blood flow around the optic disc and ensure the normal function of retinal tissue. Notably, Xu et al. recorded a significant reduction in retinal perfusion following hyperoxia, thereby supporting the adaptive regulation theory of retinal microcirculation^21^. Presently, there are few studies on the vascular density of the optic disc; thus, further studies are needed to clarify the mechanism behind it.

Our findings also showed that the axial length negatively correlated with the vascular density in the superficial and deep macula and in the peripapillary area. This finding has been confirmed in many previous studies as well^9,13,17^. With the increase in the axial length, the retinal vascular density of the myopic eyes showed a downward trend, and a series of changes occurred in the retina and the choroid. A positive correlation was also noted between the axial length and the blood flow inside the disc. This finding could be explained by the fact that the metabolic needs inside the disc may have a compensatory effect, as the retinal capillary network fails to autoregulate its blood supply in the hypoxic setting in high myopia. However, Fan et al. found no correlation between the axial length and the vascular density in the optic disc region in people aged between 18 and 50 years^22^. A possible explanation for these two contradictory conclusions is the differences in the mean SE and ages of the participants in both studies; the range of areas measured in the studies was inconsistent. Further studies that comprehensively evaluate the changes in vascular density in the optic disc area are needed. Interestingly, the present study showed a positive correlation between the SE and the peripapillary vessel density and a negative correlation with the vessel density inside the disc. However, the significance disappeared after adjustments for age, sex, and axial length. This suggests that the axial length is related to the superficial retinal vessel density, a finding that was consistent with the study by Golebiewska et al.^23^.

In a previous study, Yu et al. used OCTA to analyze participants over 35 years. They observed a significant correlation between the blood perfusion in the macular area and age^24^. Burgansky-Elias et al. also reported a negative correlation between age and flow velocity in the venules of participants over the age of 40 years^25^. However, in the present study, no correlation was found between the retinal vascular density and age. We believe that the decrease in the retinal blood flow may be more prominent in older individuals, especially those older than 35 to 40 years. Therefore, we concluded that there is no correlation between vascular density and age, possibly because the participants in the present study were between the ages of 20 and 40 years. However, further research with large samples is needed to verify this conclusion.

This study has several limitations. First, owing to the small sample size and narrow age range, the results obtained in this study cannot be generalized. Second, eyes with pathological myopia were not included in this study; thus, the results cannot reflect the changes in the retinal flow density of all highly myopic eyes. Third, compared to the spectral-domain OCTA (SD-OCTA), the swept-source OCTA (SS-OCTA) works with higher wavelengths, provides improved tissue penetration, and is more sensitive than EDI-OCT in evaluating choroidal retinopathy. In addition, SS-OCT has a wide range of displays; therefore, pathological myopia with posterior scleral staphyloma has a clearer image than SD-OCTA. However, as we mainly investigated myopia patients without pathological changes, the impact is not obvious, but we still hope to use SS-OCTA for further research^26–29^. Fourth, we did not measure the tilted optic disc. The ratio of minimum to maximum disc diameter was ≤ 0.8, which was defined as a tilted optic disc. A tilted optic disc may affect the cross-sectional area of the optic disc and increase vascular density inside the disc. However, optic disc tilt mostly occurs in people with pathologic myopia. Previous studies have shown that the degree of optic disc tilt is closely related to the severity of myopia and longer axial length^30,31^. In this study, the participants were young people without myopic maculopathy, and the highest degree was about 9.0D. Therefore, as the tilted optic disc was less, we did not make statistics. We will make further measurements in the future. Fifth, this cross-sectional study involved observing the retinal flow density in a single eye at a single time point. In the future, follow-up studies should be conducted to observe the dynamic changes in the flow density in myopic eyes to provide a reasonable explanation for the pathogenesis of myopia. Sixth, owing to the limitation in the scanning range of the OCTA device, vascular changes in areas outside the macular area and the surrounding retina were not reflected. In addition, artifacts appeared during the image acquisition process, and blood flow lower than the slowest detectable flow could not be detected^32^. Finally, OCTA has a threshold limit, which may obscure important data and lead to misinterpretation of data^33^.

In conclusion, our study showed that the superficial and deep macular vascular densities and the vascular density in the nasal RPC layer were significantly decreased in highly myopic eyes without pathological changes. However, no significant change in the blood flow in the fovea was observed, indicating that the SE does not affect the vascular density of the fovea. In addition, the axial length negatively correlated with the vascular density in the macular area and positively correlated with vascular density inside the disc. Similarly, the SE also correlated with superficial macular vascular density, although, the association with age needs to be further studied. As a practical technique for quantitative evaluation of the retinal microvascular network, OCTA imaging helps understand the underlying mechanism of pathological changes in early myopia and find potential ways to manage myopia development. The findings of this study will enable clinicians to understand better the pathogenesis of myopia and its complications.

## Methods

### Participants

This cross-sectional study was conducted in accordance with the tenets of the Declaration of Helsinki for research involving human participants. It was approved by the Medical Ethics Committee of Yijishan Hospital of Wannan Medical College. After being informed of the purpose of the research, all participants provided their written informed consent prior to the commencement of the study.

Healthy individuals with myopia were successively recruited from the Optometry Center from September 2018 to February 2019. The study participants were stratified into three groups according to the SE: mild myopia (SE, − 0.50 D to − 3.00 D), moderate myopia (SE, − 3.25 D to − 6.00 D), and high myopia (SE, ≤  − 6.00 D, without pathological changes). All the participants underwent a complete ophthalmic examination, including measurement of best-corrected visual acuity (with E-charts at a distance of 5 m) and axial length (IOLMaster, Carl Zeiss AG, Jena, Germany), slit-lamp biomicroscopy (SL-2G, TOPCON, Tokyo, Japan), non-contact tonometry (FT-100, TOMEY, Nagoya, Japan), computer optometry (TOMEY, RC-5000, Nagoya, Japan), subjective refraction (NEDEK, RT-5100, Gamagori, Japan), and OCTA (RTVue XR Avanti, Optovue, Fremont, CA, USA).

The participants were aged between 20 and 40 years, and both eyes were required to meet the following inclusion criteria: (1) binocular corrected visual acuity > 0.8; (2) intraocular pressure ≤ 21 mmHg; and (3) SE between − 0.50 D and − 6.00 D. The exclusion criteria were specified as the presence of any systemic disease; the presence of any ocular pathology such as strabismus, amblyopia, and glaucoma; and retinal and choroidal disease, for example, diffuse chorioretinal atrophy, central serous chorioretinopathy, and myopic maculopathy.

### Procedures

**Figure 4:**
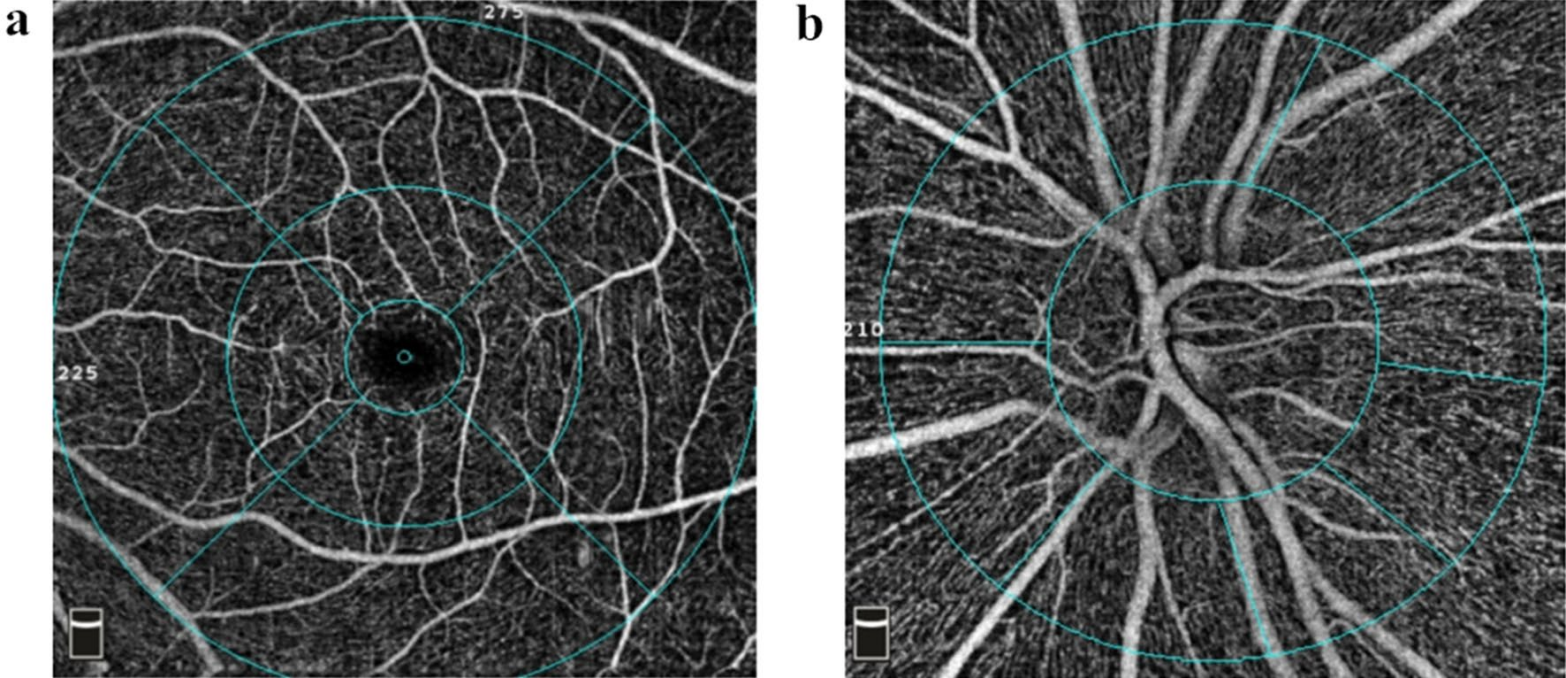
The AngioVue software (version 2017.1.0.155; Optovue Inc.) was a built-in software that provides a default angiography display protocol to define the stratification interval for en face vascular imaging (in this figure). (**a**) The microvessels in the macular area. (**b**) The peripapillary capillary network layers.

The OCTA adopts an amplitude-based imaging method, which is split-spectrum amplitude-decorrelation angiography. It performs 304 × 304 scans at a rate of 70,000 times per second to detect the blood flow based on the amplitude changes of the reflected signals of the adjacent scan sections. The AngioVue software (version 2017.1.0.155; Optovue Inc.) was a built-in software that provides a default angiography display protocol to define the stratification interval for en face vascular imaging and automatically calculate the blood flow density and surface area (Fig. 4). For the macular region, a scan is usually obtained at the parafovea and perifovea. The parafovea is an annular region consisting of a ring with a diameter of 3 mm, divided into four regions: nasal, inferior, temporal, and superior. These areas are shown on a 6 × 6 mm macular retinal blood vessel image (Fig. 4a). The superficial retinal capillary plexus is 3 μm below the internal limiting membrane to 15 μm below the inner plexiform layer (IPL), showing the retinal blood circulation around the FAZ. The boundary of FAZ area was automatically positioned, manually adjusted and delineated. The deep retinal capillary plexus is 15 to 70 μm below the IPL, showing the fine network structure. Regarding the scanning of the optic disc area, the peripapillary capillary network layer is an elliptical 700 μm ring extending from the optic disc boundary and ranging from the internal limiting membrane layer to the nerve fiber layer. It is automatically divided into eight regions by the machine's software: the nasal upper, nasal lower, inferior nasal, inferior temporal, temporal lower, temporal upper, superior temporal, and superior nasal (Fig. 4b). Due to the image amplification effect caused by axial elongation of myopia, retinal thinning, and decreased vascular density, we conducted manual correction according to the improved Bennett formula: t/s = p × q × s (q = 0.01306 × [axial length − 1.82]) where, t represents the actual value, s represents the initial measured value, p represents the magnification of OCTA, and q represents the correction factor.

### Statistical analysis

All data were analyzed and compared using the SPSS 22.0 statistical software (IBM Corp. Armonk, NY), and the measured values are expressed as mean and standard deviation. Data were analyzed using one-way analysis of variance, Pearson’s correlation coefficient, and multivariate linear regression analysis. Statistical significance was defined as P < 0.05. We statistically compared the vascular density of the superficial and deep retina in the macular region and the RPC layer in the optic disc area. We analyzed the factors that influence vascular density in these areas.

## Data Availability

The datasets generated and/or analyzed during the present study are not publicly available (obtained from the Department of Ophthalmology, Yijishan Hospital of Wannan Medical College, and the Optometry Center of Wuhu Eye Hospital) but are available from the corresponding author upon reasonable request.
